# Role of the IgM Fc Receptor in Immunity and Tolerance

**DOI:** 10.3389/fimmu.2019.00529

**Published:** 2019-03-22

**Authors:** Jun Liu, Ying Wang, Ermeng Xiong, Rongjian Hong, Qing Lu, Hiroshi Ohno, Ji-Yang Wang

**Affiliations:** ^1^Department of Immunology, School of Basic Medical Sciences, Fudan University, Shanghai, China; ^2^RIKEN Center for Integrative Medical Sciences, Kanagawa, Japan

**Keywords:** IgM, FcμR, BCR signal, humoral immune response, complement

## Abstract

Immunoglobulin (Ig) M is the first antibody isotype to appear during evolution, ontogeny and immune responses. IgM not only serves as the first line of host defense against infections but also plays an important role in immune regulation and immunological tolerance. For many years, IgM is thought to function by binding to antigen and activating complement system. With the discovery of the IgM Fc receptor (FcμR), it is now clear that IgM can also elicit its function through FcμR. In this review, we will describe the molecular characteristics of FcμR, its role in B cell development, maturation and activation, humoral immune responses, host defense, and immunological tolerance. We will also discuss the functional relationship between IgM-complement and IgM-FcμR pathways in regulating immunity and tolerance. Finally, we will discuss the potential involvement of FcμR in human diseases.

## Introduction

B cells produce different classes of antibodies (Ab), including IgM, IgD, IgG, IgA, and IgE. Ab constitutes a variable F(ab) region that binds to antigen (Ag) and a constant Fc region that mediates effector function. Cellular receptors for the Fc region mediate a variety of functions including phagocytosis of Ab-opsonized pathogens and induction of cellular cytotoxicity. Recent studies have unveiled three Fc receptors for IgM, including Fcα/μ receptor (Fcα/μR), polymeric immunoglobulin receptor (pIgR), and Fcμ receptor (FcμR). Fcα/μR, pIgR, and FcμR are all type I transmembrane proteins belonging to the immunoglobulin (Ig) gene superfamily. Fcα/μR is expressed by both hematopoietic and non-hematopoietic cells (1, 2), and has been shown to play an important role in humoral immune responses, especially in pro-inflammatory functions of marginal zone B cells in sepsis (3). pIgR is expressed on the basolateral surface of ciliated epithelial cell in the mucosal epithelium (4, 5), but not in hematopoietic cells (6). The main function of pIgR is to transport dimeric IgA and polymeric IgM from the lamina propria across the epithelial barrier to mucosal surfaces (7). FcμR was discovered relatively recently and its function has not been fully elucidated. Here we summarize the results of FcμR published over the past several years, and discuss how it contributes to immunity and tolerance.

## Molecular Characteristics of FcμR

The existence of a receptor for IgM was noted more than 40 years ago (8–16). Biochemical analysis revealed that human FcμR had a molecular weight of ~60-kDa (17). Molecular cloning of *FCMR*, the gene encoding human FcμR, revealed that it is a single copy gene located on chromosome 1q32.2, adjacent to two other IgM associated Fc receptor genes, polymeric Ig receptor gene (*PIGR*) and the gene of FcR for IgA and IgM (*FCAMR*) (18). Human FcμR is a type I transmembrane protein of 390 amino acids (aa), composed of a 234-aa extracellular domain, a 21-aa transmembrane segment, and a 118-aa cytoplasmic tail (19, 20). BW5147 T cells ectopically expressing human FcμR exhibited specific binding to IgM but not any other Ab isotypes, demonstrating that FcμR is the bona-fide receptor for IgM (18). Unlike many other FcRs, the cytoplasmic tail of human FcμR does not contain any immunoreceptor tyrosine-based activation (ITAM) or inhibitory (ITIM) motifs. Instead, it contains conserved serine and tyrosine residues, which match the recently described Ig-tail tyrosine (ITT) motif (21, 22). Crosslinking human FcμR with either anti-FcμR monoclonal antibodies or preformed IgM immune complexes triggered the phosphorylation of these serine and tyrosine residues in FcμR-overexpressing BW5147 T cells, suggesting that FcμR could serve as an ITT phosphorylation molecule to interact with and influence the B cell receptor (BCR) signaling (23). Human FcμR is predominantly expressed by B, T, and NK cells, but not by monocytes, granulocytes, erythrocytes, and platelets (18). Human FcμR binds more efficiently to the Fc portion of IgM reactive with surface proteins than to the Fc portion of free IgM (24), suggesting that FcμR might modulate the signal of B, T, and NK cell surface receptors or proteins recognized by natural or immune IgM.

The mouse FcμR gene (*Fcmr*) is also a single copy gene located on chromosome 1 (56.89 cM), adjacent to *Pigr* and *Fcamr* (25). Although mouse and human FcμR have similar molecular structure, they share only 54% aa identity. Mouse FcμR also specifically binds to IgM (25, 26). Unlike human FcμR, we found that mouse FcμR is predominantly expressed in B lymphocytes by both microarray of a panel of immune cell types and FACS analyses (25, 27, 28). However, others have reported that monocytes, macrophages, granulocytes, and dendritic cells also express FcμR (29, 30). The expression levels of Mouse FcμR are different among different B cell subsets. The hierarchy of FcμR levels on various B cell subsets is as follows: marginal zone precursor (MZP, IgM^hi^CD21^hi^CD23^hi^) > follicular B (FOB, IgM^lo^CD21^lo^CD23^hi^) > marginal zone B (MZB, IgM^hi^CD21^hi^CD23^lo^) > newly formed B (CD93^+^CD21^−^CD23^−^) cells (28, 31). FcμR expression level is indistinguishable between B1 (CD5^+^) and B2 (CD5^−^) cells in the spleen. In the peritoneal cavity, FcμR expression level in each B cell subsets follows the order: B2 (CD11b^−^CD5^−^) ≅ B1a (CD5^+^) > B1b (CD11b^+^CD5^−^) cells (31, 32). In addition, FcμR expression is very low in pro-B (B220^+^CD43^+^) and pre-B (B220^+^CD43^−^IgM^−^) cells, and slightly upregulated in immature B cells (B220^dull^IgM^+^) in the bone marrow (BM) (27, 31, 33). FcμR expression in the germinal center (GC) B cells (CD95^+^GL7^+^) is much lower than that in naïve B cells (27), suggesting that FcμR is down-modulated during GC reaction. FcμR is expressed at higher levels in plasmablasts compared to plasma cells. Intriguingly, FcμR is also expressed by IgG- or IgA-positive B cells, suggesting that it may play a role in switched B cells (32).

It is intriguing that genes encoding FcμR, Fcα/μR, and pIgR are located in the same chromosomal region (18, 25), suggesting that these genes are evolutionarily related and might have derived from a common ancestor gene. However, in contrast to FcμR which only binds to IgM, Fcα/μR binds both IgM and IgA (3, 34, 35). Moreover, pIgR binds both IgM and IgA via their associated J chains and is essential for the transcytosis of polymeric IgA and IgM to the gut (36). The expression pattern is also quite different among these receptors. FcμR is predominantly expressed by B cells in mice and by B, T, and NK cells in humans (18, 25). In contrast, Fcα/μR is expressed by macrophages, B cells, intestinal lamina propria and several other cell types (35), and pIgR is mainly expressed on the intestinal epithelial cells (4, 5). Although FcμR was originally designated as Fas apoptotic inhibitory molecule 3 or TOSO (37), it is now clear that both human and mouse FcμR have no inhibitory activity against Fas-mediated apoptosis (38, 39).

## FcμR in B Cell Development and Maturation

Several *Fcmr*-deficient (KO) and B-cell-specific deletion of *Fcmr* (BKO) mouse strains have been generated. (1) We and Kubagawa et al. share the constitutive FcμR knockout strain (*Fcmr*^tm1Ohno^) in which exons 2–4 were deleted in 129/Sv ES cells and the mutant mice had been backcrossed to C57BL/6 mice for > 12 generations. The neo gene used in drug selection was removed by crossing with Cre-Tg mice (27, 28, 32, 40–42); (2) Mak et al. and Coligan et al. share the constitutive FcμR knockout strain (*Fcmr*^tm1Mak^) where exons 2–8 were deleted in 129/Sv ES cells and the mutant mice had been backcrossed to C57BL/6 mice. The neo gene remained in the targeted allele (29–31, 43, 44); (3) Lee et al. have the constitutive FcμR knockout strain (*Fcmr*^tm1.2Khl^) and a strain with floxed *Fcmr* allele, with exons 4–7 were deleted or flanked by loxP sites, respectively. No neo gene remained in the targeted allele and both mice are on a pure B6 background (45–47); Baumgarth et al. generated the *Fcmr*^flx/flx^*Cd19*-Cre^+^ strain in which exon 4 was deleted by CD19-driven Cre. The mutant mice are on a pure B6 background (33, 48). A comparison of the phenotypes of *Fcmr*^−/−^ mice generated and/or analyzed by different groups is shown in Table 1.

**Table 1:** Comparison of the phenotypes of *Fcmr*^−/−^ mice generated/analyzed by different groups.

**Mouse strain**		***Fcmr***^****tm1Ohno****^		***Fcmr***^****tm1Mak****^		***Fcmr*^**tm1.2Khl**^**	***Fcmr*^**flx/flx**^ CD19-Cre+**
Targeting strategy		Exons 2–4 were deleted in 129/Sv ES cells and the mice backcrossed to C57BL/6 mice. The neo gene was removed		Exons 2–8 were deleted in 129/Sv ES cells and the mice backcrossed to C57BL/6 mice. The neo gene was not removed		Constitutive FcμR knockout strain and a conditional knockout with exons 4–7 deleted. Pure B6 background	Exon 4 was deleted by CD19-driven Cre. Pure B6 background
Research group		Hiromi Kubagawa	Ji-Yang Wang	John E. Coligan	Tak W. Mak	Kyeong-Hee Lee	Nicole Baumgarth
Related references		(32, 41)	(27, 28, 40)	(31)	(29, 30, 44)	(45, 47)	(33, 48)
B & T cells	BM	Pro-B, Pre-B, Immature B, Recirculating B	Pro-B, Pre-B, Immature B, Recirculating B	Pro-B, Pre-B, Immature B, Recirculating B			Pro-B, Pre-B, Immature B, Recirculating B, B1
	Spleen	Total T, Total B, FOB, Newly formed B, Regulatory B, MZB, B1	Total T, Total B, FOB, T3, MZB	Total B, Newly formed B, Regulatory B, MZB, B1, FOB		Total T, Mature B, Newly formed B, Regulatory B, MZB, B1a,	Total B, FOB number, Newly formed B, MZB number, MZB ratio, B1, B1a, GCB
	PC	Total T, Total B, B1a, B1b, B2	B1a	Total B, B1a, B1b, B2		B1a, B1b	
B cell function			BCR-triggered Ca^2+^ influx, antigen presentation, CSR, B cell survival induced by BCR cross-linking, BCR-triggered activation of non-canonical NF-κB pathway	B cell survival induced by BCR cross-linking		B cell activation	Turnover and survival of B cells
Homeostasis & Humoral immune responses	Basal Ig levels	IgG2b, IgG2c, IgA, IgM, IgG3	IgG3, IgG2b IgG2c, IgA, IgM	3 month old: IgM, IgG3, IgG2b IgG2a, IgA, IgG1; 6 month old: IgM, IgG1, IgG2b IgG2a, IgG3, IgA			IgG, IgA, IgM
	TI response	Phosphorylcholine response	TI-1 & TI-2 responses, MZB response to LPS	GCB & PC	Response to LPS		
	TD response	Affinity maturation of Abs, primary IgG1 and secondary IgM anti-CGG responses	GC formation, Memory B and plasma cell, Ab production in primary and secondary responses	GCB, PC, IgM, IgG2a			
Infectious immunity		Low dose of R36A: increased IgM and IgG3 responses; High dose of R36A: no increase	*C. rodentium*-induced sepsis		*Listeria*-induced & persistence-prone infection	TNFα-mediated liver damage, Influenza virus infection	Influenza virus infection
B cell tolerance		IgM and IgG anti-dsDNA, ANAs; Serum auto-antibody titers and Mott cell formation in FcμR KO B6/lpr mice but no lupus-like nephritis	IgG anti-dsDNA Abs, rheumatoid factor, ANAs	IgG anti-dsDNA and ANAs	EAE	Differentiation/maintenance of regulatory B cells, IgM an IgG anti-dsDNA, or anti-ssDNA	IgM and IgG anti-dsDNA

*Black: not affected*.

Blue: increased or enhanced.

Red:decreased or impaired.

*Blank: not investigated*.

B cell development proceeds from pro-B, pre-B to immature B cells in BM (49). Immature B cells then migrate to the periphery where they further differentiate into various mature B cell subsets that play distinct roles. The survival and maturation of B cells are dependent on the strength of tonic BCR signal (50, 51). Studies from our group, Honjo et al. and Nguyen et al. revealed that FcμR deficiency did not significantly affect B cell development, but altered the numbers of different B cell subsets (32, 33). We and Honjo et al. found that MZB were severely reduced in KO mice (27, 32) whereas Nguyen et al. found decreased proportion of MZB but the absolute numbers of MZB were not affected (Table 1) (33). Honjo et al., Choi et al., and Nguyen et al. reported that the splenic B1 cells were increased in KO mice (31–33). More recently, we found reduced tonic BCR signaling in FcμR-deficient MZB, which we think led to their decreased numbers in KO mice (28). In contrast, Honjo et al. suggested that the reduction of MZB in KO mice was due to their rapid differentiation into plasma cells (41). Lee et al. found decreased numbers of B cells in the spleen and lymph nodes (47). Choi et al. found that B-1a were increased but B-2 were decreased in the peritoneal cavity and that FOB were decreased in the spleen (Table 1) (31), which were similar to the phenotypes found in *S*μ^−/−^ mice that lack secreted IgM (52, 53). Taken together, these results indicate that FcμR affects the maturation or differentiation of various B cell subsets.

## FcμR in B Cell Survival and Activation

We found that FcμR cell surface expression was upregulated after BCR cross-linking with anti-IgM Abs but only moderately increased by CD40L or LPS stimulation under *in vitro* culture conditions (40). Choi et al. reported that FcμR transcript levels were markedly reduced by stimulation of spleen B cells with anti-IgM, LPS or anti-CD40 (31), suggesting that FcμR expression is regulated at both transcriptional and posttranscriptional levels. Moreover, we and others demonstrated that FcμR specifically enhanced B cell survival induced by anti-IgM stimulation (Table 1) (27, 31, 40). Immunofluorescence and co-immunoprecipitation revealed physical interaction between FcμR and BCR on the plasma membrane of primary B cells (40). Although FcμR deficient B cells exhibited normal Ca^2+^ influx after BCR crosslinking, their survival was reduced compared with WT B cells (27), indicating that FcμR did not affect the early BCR signaling event such as Ca^2+^ influx but affected the late response such as B cell survival. Analysis of signaling molecules downstream of BCR revealed that FcμR promoted the activation of the non-canonical NF-κB pathway and the induction of BCL-xL (40). These results suggest that FcμR and BCR cooperate in signal transduction to promote B cell survival. FcμR does not contain any ITAM motifs but instead contains several conserved tyrosine and serine residues in its cytoplasmic tail (19, 20, 23, 26). A detailed mutational analysis has revealed that the tyrosines 315, 366, and 385 are not required for ligand (IgM) binding. However, tyrosine 315, as well as the entire intracellular domain, was shown to be required for inhibiting an IgM anti-FAS Ab-induced apoptosis (24). It remains to be investigated how FcμR specifically affects the late phase of BCR signaling and whether these tyrosine and serine residues are involved.

**Figure 1:**
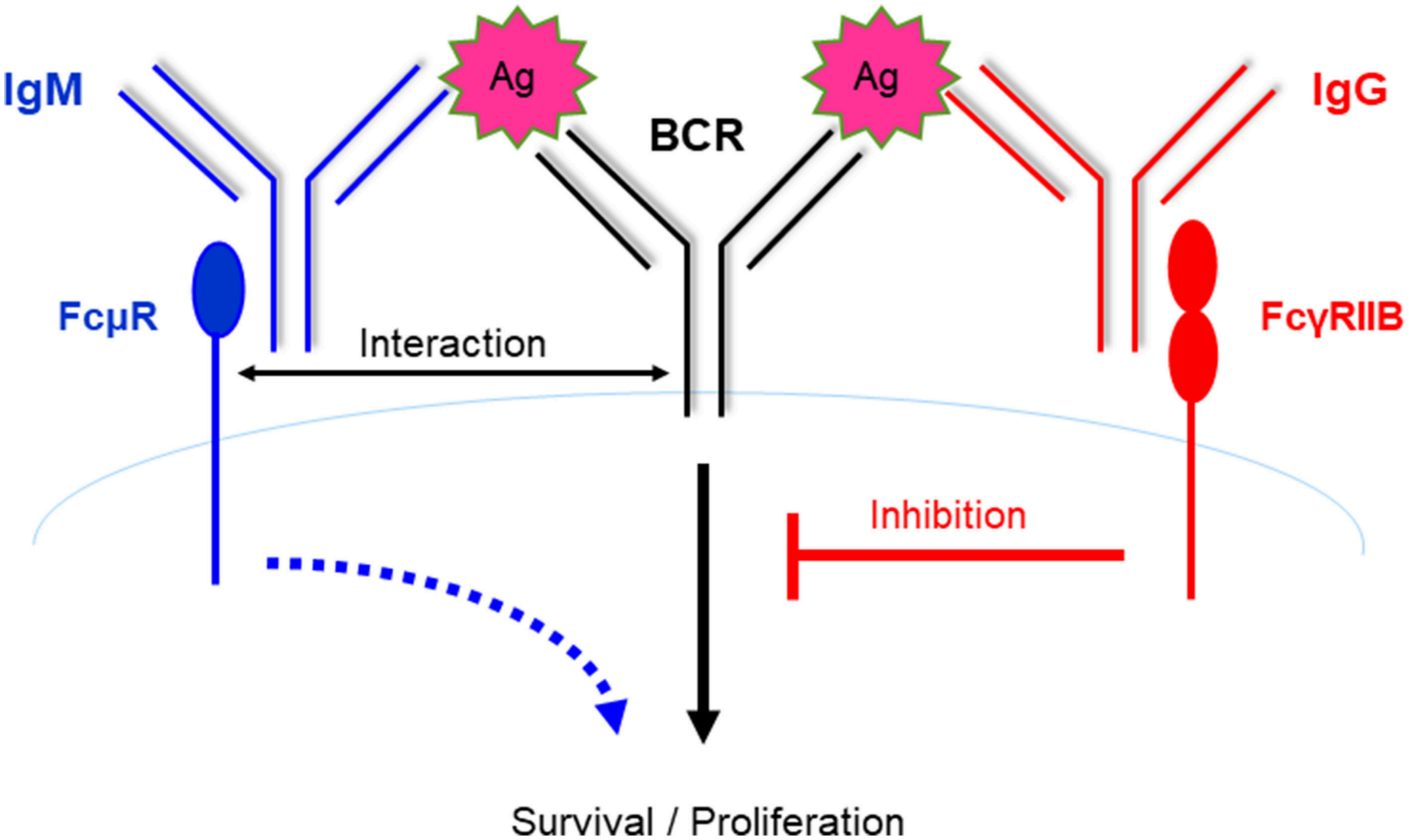
B cells express two types of Fc receptors of opposing functions. FcμR promotes B cell activation via interacting with the BCR and potentiating BCR signaling. In contrast, B cells express FcγRIIB, which inhibits B cell activation upon binding to immune complexes containing IgG and the cognate Ag, which then results in colligation of FcγRIIB and the BCR. Therefore, B cells express two types of Fc receptors of opposing functions. It is suggested that these two receptors function in a spatial-temporal manner to positively and negatively regulate B cell activation during humoral immune responses (see Figure 2).

It is well-known that B cells express FcγRIIB, which inhibits BCR signaling and B cell activation upon binding IgG-Ag immune complexes, which then results in colligation of FcγRIIB and the BCR. Therefore, B cells express two types of Fc receptors, FcμR and FcγRIIB, which promotes and inhibits BCR signaling and B cell activation, respectively (Figure 1). More recently, Nguyen et al. reported that FcμR limited tonic BCR signaling in immature B cells by regulating the expression of IgM BCR (33). Therefore, FcμR regulates both the cell surface expression and the function of BCR.

## Role of FcμR in Humoral Immune Responses

The basal Ig levels reflect the immune homeostasis at the steady state. We found that basal serum IgM levels were elevated in the absence of FcμR in a gene dosage-dependent manner, suggesting that a portion of the serum IgM actually binds to the FcμR in WT mice (27). Nguyen et al. found the same results and attributed the high IgM level to the elevated numbers and hyper-activation of B1 cells in the spleen (33). In addition, Honjo et al. found that IgM levels were elevated and that the IgG3 levels were slightly elevated in KO mice (32). In contrast, Choi et al. reported that only IgG1 levels were reduced in 3-month old mice and IgG3 and IgA levels were slightly elevated in 6-month old mice (31). Therefore, FcμR-deficient mice generated by different groups all exhibited increased levels of serum IgM and/or IgG3 (Table 1). These results implicate a role for FcμR in B cell homeostasis.

**Figure 2:**
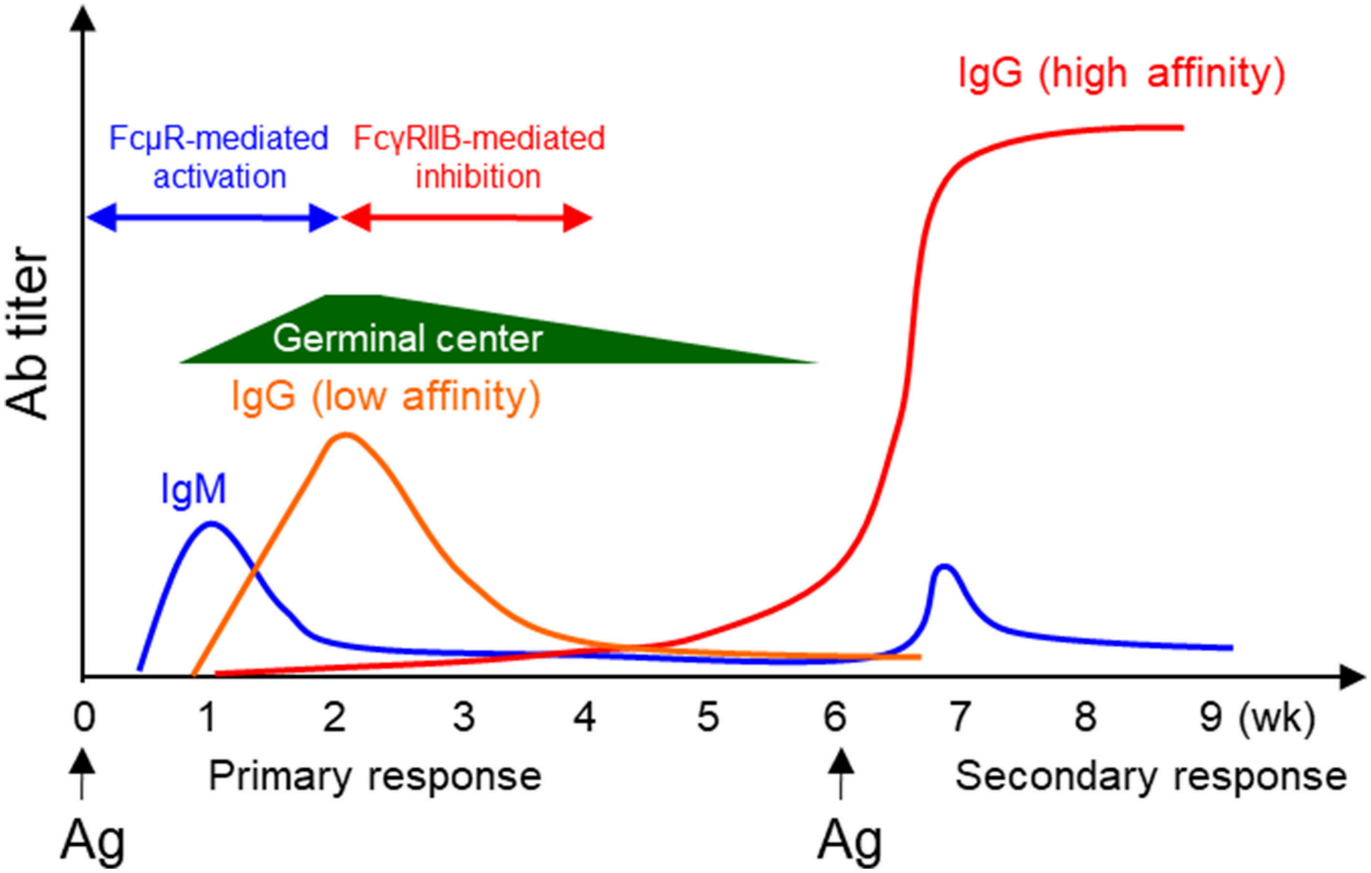
Positive and negative regulation of humoral immune responses by FcμR and FcγRIIB. During a typical T-D humoral immune response, Ag-specific IgM is produced first, followed by IgG production. Based on the results that FcμR promotes B cell activation and Ab production and the earlier findings that FcγRIIB inhibits B cell activation and Ab production, we propose an autoregulatory mechanism for T-D humoral immune responses. During the early phase of the response, B cell activation is enhanced by FcμR-mediated positive signals. However, at a later phase of the response, further B cell activation is suppressed by FcγRIIB-mediated inhibitory signal.

We found that KO mice had significantly decreased production of NP-specific IgG1 during both primary and secondary responses against a T-dependent (T-D) Ag, NP-CGG (27, 28), likely due to impaired GC formation and reduced memory and plasma cell differentiation. Similarly, Honjo et al. found impaired primary IgG1 and secondary IgM anti-CGG responses, but normal Ab affinity maturation (32). During humoral immune responses to T-D Ag, Ag-specific IgM is first produced, which is followed by the production of Ag-specific IgG. Based on our results that FcμR is required for efficient Ab production and the earlier findings that FcγRIIB inhibits B cell activation and Ab production, we propose an autoregulatory mechanism for T-D humoral immune responses [(27) and Figure 2]. During the early phase of the response, when the amount of Ag-specific IgM is greater than that of Ag-specific IgG, B cell activation is enhanced by FcμR-mediated positive signals. However, during the later phase of the response, when the amount of Ag-specific IgG is greater than that of Ag-specific IgM, further B cell activation is suppressed by FcγRIIB-mediated inhibitory signal (Figure 2). B cell activation and Ab production can thus be positively and negatively regulated by Ag-specific IgM and IgG present in the local environment, respectively.

Consistent with the reduced survival in FcμR-deficient B cells after BCR crosslinking, FcμR KO mice had decreased Ab production against a type 2 T-independent (T-I) Ag, NP-FICOLL (27), since response to this type of Ag is largely dependent on BCR signal. Additionally, we found that FcμR KO mice had impaired Ab production against a type 1 T-I Ag, NP-LPS (28), which activates B cells through both BCR and toll-like receptor 4. Moreover, we found that MZB in KO were not activated upon LPS injection (28). Since MZB cells are thought to participate in the response to LPS, the reduced Ab production to NP-LPS immunization could be due to both a reduction in the number of MZB cells and their impaired response to LPS. Our results are consistent with the earlier finding by Lang et al. that FcμR-deficient mice had reduced LPS response *in vivo* (29). Choi et al. found elevated numbers of GC B cells and accelerated plasma cell formation during type 1 and 2 T-I immune responses and secondary T-D immune responses (31). In addition, the plasma cell formation in primary T-D immune response was also increased (summarized in Table 1). The reason for the discrepancies among results from different groups is unclear but could in part be attributable to the differences in the targeting strategy, the immunization protocol, and the genetic background as well as rearing environment of these mutant mice. Collectively, these results suggest that FcμR regulates humoral immune responses.

## FcμR in Infectious Immunity

As summarized in Table 1, FcμR-deficient mice generated a higher titer of anti-phosphorylcholine Ab and a lower titer of anti-protein Ab than did WT mice when infected with a low dose of live non-encapsulated strain of *Streptococcus pneumoniae* (R36A) (32). However, a high dose of pathogen infection induced no significant difference in Ab production between WT and KO mice. We found that FcμR protected mice against sepsis induced by *Citrobacter rodentium*, a gram-negative bacterium that has LPS on the outer membrane (28). Similarly, Lang et al. found that the absence of FcμR resulted in limited cytokine production after *Listeria monocytogenes* (a gram-positive bacterium) infection and increased death of the infected KO mice (29). They also found that FcμR was required for the control of persistence-prone virus infection in a lymphocytic choriomeningitis virus model system (44). In addition, Yu et al. reported that FcμR deficiency resulted in increased numbers of IL-10–producing B cells, which mediated regulation of T cell immunity during influenza infection (45). On the contrary, Nguyen et al. found that FcμR expression on B cells, but not Fcα/uR expression or complement activation, was important for the antiviral IgG responses (48). B cell-specific KO mice lacked robust clonal expansion of influenza hemagglutinin-specific B cells early after infection and developed fewer IgG plasma cells and memory B cells in the spleen and BM, compared with WT mice (48). These results suggest that FcμR has important roles in B cell responses to protein and non-protein determinants of live pathogens and in cooperating with other immune cells to protect the mice against infection.

## FcμR in B Cell Tolerance

**Figure 3:**
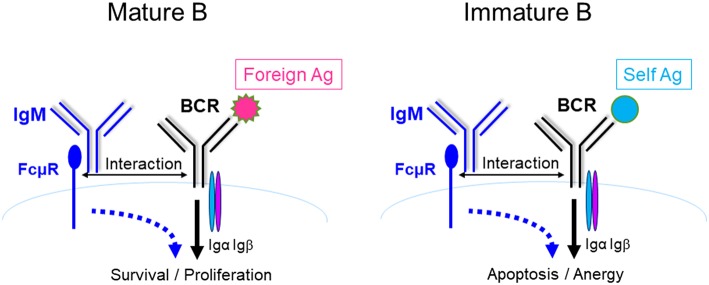
A model for FcμR-mediated immunity and tolerance. FcμR promotes the survival and activation of mature B cells by interacting with the BCR and potentiating foreign Ag-triggered BCR signaling **(left)**. By analogy, FcμR might also promote self Ag-triggered BCR signaling in immature B cells and contribute to the deletion/anergy of autoreactive immature B cells in the BM **(right)**. Ag-specific IgM/IgG are illustrated in the scheme shown in Figures 1, 2 and 4 to suggest that those reactions occur during an immune response. In contrast, IgM shown in this scheme is not Ag-specific to implicate that these reactions can occur in the absence of Ag-specific IgM.

B cell central tolerance ensures autoreactive immature B cells to undergo clonal deletion, anergy or receptor editing while peripheral tolerance functions to delete autoreactive B cells generated during GC reaction. We and others found that KO generated autoreactive antibodies including anti-dsDNA, rheumatoid factor, and anti-nuclear antibodies (27, 32, 33, 41, 45). Honjo et al. crossed FcμR-deficient mice with the Fas-deficient autoimmune-prone B6.MRL *Fas*^lpr/lpr^ mice (B6/lpr), and found that the double mutant mice had accelerated development of autoreactive Ab including anti-dsDNA and anti-Sm Ab (41). They also found enhanced formation of Mott cells, aberrant plasma cells which accumulate large amount of Ig in the rough endoplasmic reticulum, in KO mice. Nevertheless, KO mice with autoimmune-prone background have normal kidney function and equal mortality compared to control group (41). Brenner et al. reported that KO mice were protected from the development of severe experimental autoimmune encephalomyelitis (EAE), a mouse model for human multiple sclerosis. Their results suggested that FcμR regulated the function of dendritic and regulatory T cells (30). Collectively, a common feature of KO and BKO generated by different groups is the production of various autoantibodies (Table 1). It remains to be investigated how FcμR regulates B cell tolerance. We have shown that FcμR promotes B cell survival and activation by interacting with BCR and potentiating Ag-triggered BCR signaling (Figure 3, left panel). By analogy, we think that FcμR might also promote self Ag-triggered BCR signaling in immature B cells and contribute to the deletion/anergy of autoreactive immature B cells in the BM (Figure 3, right panel). Further studies are required to clarify whether and how FcμR contributes to B cell central or peripheral tolerance.

## Functional Relationship Between IgM-Complement and IgM-FcμR Pathways

**Figure 4:**
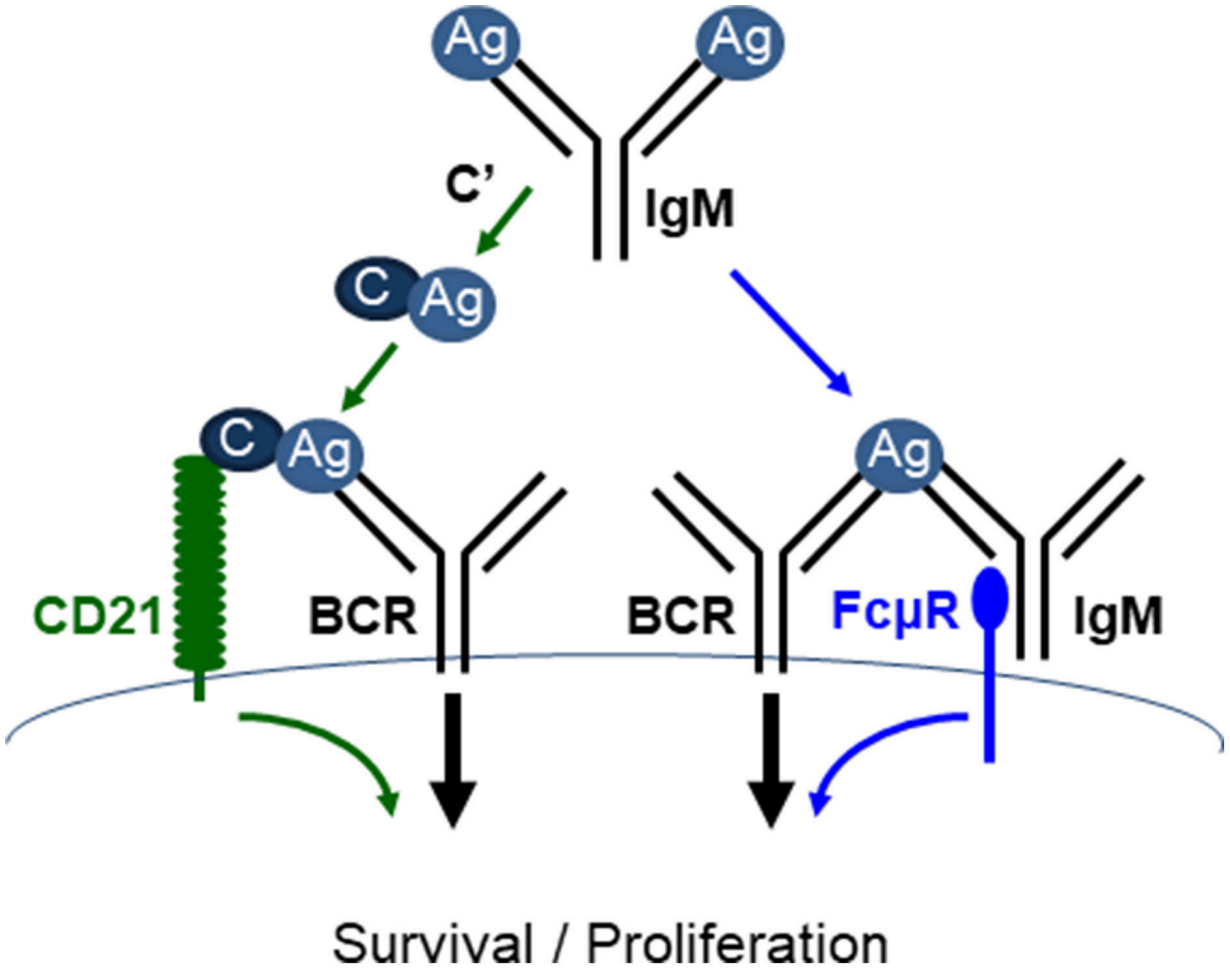
FcμR and complement receptor promote mature B cell survival and activation. IgM (pentamer; for simplicity a monomeric IgM is depicted) binds to antigens (Ag) and the resulting IgM-Ag complexes can enhance B cell survival and activation through at least two pathways. (1) IgM-Ag complexes can activate complement (C') cascade, resulting in C' fixation on the Ag, which can crosslink BCR and C' receptor (CD21) on B cells (green arrows). (2) IgM-Ag complexes can crosslink BCR and FcμR on B cells (blue arrows). It remains to be elucidated whether these two pathways function cooperatively, independently or competitively [adapted from Ouchida et al. (27)].

IgM is the first Ab to appear during evolution and the only isotype produced by all species of jawed vertebrates (54–56). It is also the first isotype produced during a T-D immune response and is the first line of host defense (57). IgM is not only an effector molecule, but also regulates humoral immune response. Earlier studies suggested that IgM promotes the production of antigen-specific IgG via activating complement. However, a recent study by Heyman's group demonstrated that mice expressing a mutant IgM unable to activate complement (Cμ13) had completely normal humoral immune responses (58), thus raising the possibly that in addition to complement activation, there are alternative pathways by which IgM elicits its function. As discussed above, IgM can elicit its function through FcμR. Therefore, both IgM-FcμR and IgM-complement pathways function to regulate B cell survival and activation (Figure 4). It remains to be investigated whether these two pathways function cooperatively, independently, or competitively.

## FcμR in Human Diseases

Human FcμR was shown to be overexpressed and associated with the anti-apoptotic characteristic in chronic lymphocytic leukemia (CLL) (59, 60). CLL is a malignancy of mature IgM^+^ B cells that exhibit features of polyreactive, partially anergized B cells related to memory B cells (60). Several studies showed that *FCMR* expression in CLL was significantly higher than that in healthy controls and other B cell lymphoproliferative diseases (59, 61–63). In addition, CLL patients also had higher serum titers of FcμR compared with healthy donors. The serum FcμR, a 40-kDa soluble form of the receptor generated by alternative splicing, was produced by both CLL B and non-CLL B cells (64). Cox regression analysis indicated that high expression of *FCMR* was an independent indicator for shorter treatment-free survival in CLL (64). Thus, FcμR is associated with the disease progression and patient survival and may serve as a prognostic factor. Interestingly, FcμR can even be used as a target for a more selective treatment of CLL by T cells expressing a chimeric antigen receptor (CAR-T), and initial studies have implicated a superior therapeutic index with anti-FcμR CAR-T cells for the treatment of CLL compared with the currently used therapies (65).

The reason that causes FcμR upregulation in CLL remains unclear. A negative correlation was observed between age and FcμR expression (59). In addition, overexpression of FCMR seemed to promote the chromosomal abnormalities (61). These shreds of evidence suggest that FcμR expression is related to the degree of genomic activity. Intriguingly, surface FcμR levels were also significantly elevated in the non-CLL B cells and T cells, suggesting that abnormal expression of FcμR is associated with systemic gene regulation (64). FcμR expression is significantly upregulated by BCR stimulation but decreased by CD40 ligation, which suggested that autoreactive BCR signaling as a key mediator of apoptosis resistance in CLL (63). Besides, FcμR expression on CLL cells is downmodulated at both the mRNA and protein levels by TLR7 and TLR9 agonists (60). This study also revealed that FcμR not only localized to the cell membrane but also accumulated in the trans-Golgi network (60). FcμR may internalize IgM-Ag complexes and thus serve as a receptor for the delivery of therapeutic Ab–drug conjugates into CLL cells (60). In addition, based on the findings in mice, human FcμR may have some roles in TNFα-mediated liver damage (47), malaria vaccine promotion (46), and the function of pancreatic islets (66).

## Conclusion

IgM is an old immunoglobulin isotype, which can bind to Ag with high avidity and activate the complement cascade. Its authentic and specific Fc receptor (FcμR) is the last one to be explored after Fcα/μR and pIgR. Although there are some discrepancies regarding the function of FcμR published by different groups, the following common abnormal phenotypes have been observed: (1) alterations in B cell maturation and differentiation; (2) impaired humoral immune responses; (3) autoantibody production. In addition, FcμR appears to contribute to the initiation/progression of human CLL and has recently been tested as a therapeutic target for treating CLL. Yet still many questions remain to be answered, including the function of FcμR in the generation, maintenance and activation of memory B cells, and in host defense mediated by natural IgM produced by B-1 and Ag-specific IgM produced by B-2 cells. Further studies are required to fully uncover the function of FcμR in immunity and tolerance.

## Author Contributions

JL provided a draft of the manuscript. YW completed the references. EX and RH provided all the figures. QL revised the manuscript. HO corrected the manuscript. J-YW designed the outline and made the final corrections of the manuscript.

### Conflict of Interest Statement

The authors declare that the research was conducted in the absence of any commercial or financial relationships that could be construed as a potential conflict of interest.
